# Data on the *in vitr*o elution of substances from three types of polysulfone membrane dialyzers as well as a non-polysulfone cellulose triacetate membrane dialyzer evaluated using ultraviolet absorption

**DOI:** 10.1016/j.dib.2021.107490

**Published:** 2021-10-19

**Authors:** Yoshinori Sato, Hayato Horiuchi, Shinji Fukasawa, Shingo Takesawa, Jun Hirayama

**Affiliations:** a Department of Clinical Engineering, Faculty of Health Sciences, Komatsu University, Ishikawa, Japan; b Department of Medical Engineering, National Center for Child Health and Development, Tokyo, Japan; c Department of Medical Engineering, Kyushu University of Health and Welfare, Miyazaki, Japan

**Keywords:** Dialyzer, Hemodialysis, Ultraviolet absorption, Polyvinylpyrrolidone

## Abstract

We evaluated the influences of the priming process (washing with saline), saline circulation conditions, and saline incubation on the *in vitro* elution of substances from three types of polysulfone (PSu) membrane dialyzers sterilized using gamma irradiation [NV-15X (Toray Industries, Inc.)], autoclaving [RENAK-PS1.6 (Kawasumi Laboratories, Inc.)], or in-line steam [FX-140J (Fresenius Medical Care)] methods as well as a non-PSu cellulose triacetate (CTA) membrane dialyzer [FB-150U(NIPRO)]. The effect of priming was evaluated by circulating 1000 mL of saline through the dialyzers at a rate of 100 mL/min and measuring the elution level of the substances by determining their ultraviolet (UV) absorption at 220 nm using spectrophotometry. All the tested dialyzers showed that the elution of the substances decreased as per the order of sample collection. Primed dialyzers were used in the subsequent experiments. Circulating saline through the primed membrane dialyzers at a flow rate of 100 mL/min caused time-dependent elution of substances from all the tested dialyzers; increasing the flow rate to 200 mL/min did not have a significant effect on the time-dependence or elution amount at each time point (0–8 h). The elution was also evaluated after incubating the membrane dialyzers with saline for 24 h. A co-submitted article (Sato et al., 2021) detailed the preparation of the identical experimental circuits, as well as the influences of saline washing, saline circulation conditions, and saline incubation on the elution of the hydrophilic agent polyvinylpyrrolidone (PVP) from each dialyzer using the Müller method, which can enable specific detection of PVP (Müller, 1968). The relative elution levels of PVP among the dialyzers and the experimental conditions were different from those of substances determined using UV (220 nm) absorption. Our data might be used for further development of experiments for identifying non-PVP substances eluted from dialyzers by providing information regarding the conditions of the elutions and types of dialyzers from which they are eluted.

## Specifications Table

**Table:** 

Subject	Hematology
Specific subject area	*In vitro* characterization of hemodialyzer membranes
Type of data	Graph Figure Table
How data were acquired	The UV (220 nm) absorption of the eluates was determined using a spectrophotometer (Hitachi; U-5100). The statistical analysis was conducted with the Statcel 4 (The Publisher OMS Ltd. Japan).
Data format	Raw
Parameters for data collection	1. The flow rates at which saline was circulated in the experimental circuits. 2. Duration of the circulation.
Description of data collection	Each dialyzer was set as per the experimental circuits described in Fig. 1A–C of the co-submitted manuscript [1]. In experiments with the circuits in Fig 1A and B, saline was circulated in the experimental circuit at a rate of 100 mL/min or 200 mL/min and was then collected for the measurement of UV (220 nm) absorption. In experiments with the circuit in Fig. 1C, each dialyzer was incubated with saline at 25 °C for 24 h without circulation. Following incubation, the saline was recovered for the measurement of UV (220 nm) absorption.
Data source location	Komatsu University Komatsu-city, Mukaimoto-ori-machi, Japan
Data accessibility	With the article
Related research article	Y Sato, H Horiuchi, S Fukasawa, S Takesawa, J Hirayama. Influences of the priming procedure and saline circulation conditions on polyvinylpyrrolidone *in vitro* elution from polysulfone membrane dialyzers. Biochemistry and Biophysics Reports. Accepted.

## Value of the Data

• In this study, when the UV (220 nm) absorption of the eluted substances was measured, the elution patterns of the substances from the dialyzers (Table 1) were distinct from those determined using the Müller method [1], which can specifically detect PVP [2]. This fact suggests that substances other than PVP are eluted from these dialyzers.
• Although PVP reportedly absorbs UV light [3], this study provided evidence indicating that the evaluation of the PVP elution from dialyzers is not feasibly performed by measuring the UV (220 nm) absorption. This idea is in agreement with a recent report as per which, the detection of the UV absorption spectrum of the PVP is required to determine the concentration of the PVP eluted from dialyzers [4].
• Several studies have focused on PVP elution from PSu membrane dialyzers because PVP could cause adverse effects in the human body [5], [6], [7], [8], [9], [10], [11]. Our data provides evidence that substances other than PVP can be eluted from PSu membrane dialyzers, emphasizing the importance of identifying the eluted substances for ensuring the safety of hemodialysis patients.
• Researchers and medical staff working on biomedical substances, such as dialyzer membranes, can benefit from our data.
• Our data could be used for developing experiments designed to identify the additional substances eluted from dialyzers by providing information regarding the conditions of the elutions and the types of dialyzers from which they are eluted.

## 1 Data Description

**Fig. 1:**
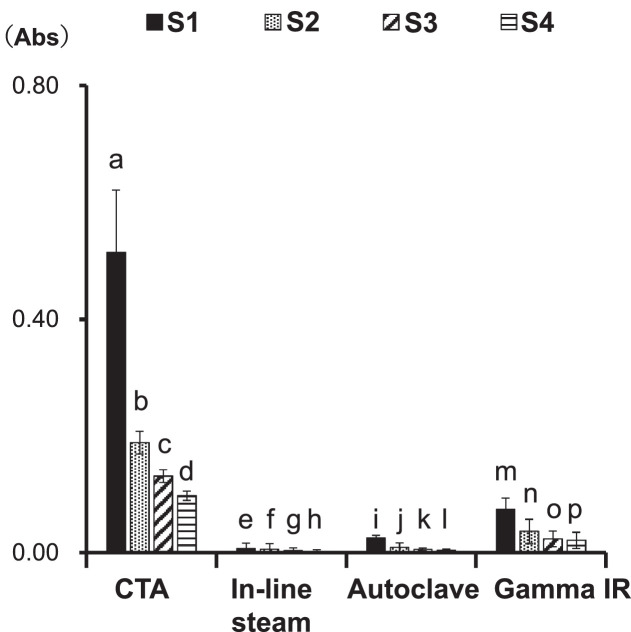
Profiles of *in vitro* elution of unidentified substances from dialyzers by washing them with saline.

Each dialyzer was set in the circuit described in Fig. 1A of the co-submitted article [1]. Thereafter, 1000 mL of saline was circulated in the experimental circuit at a flow rate of 100 mL/min and was collected from the circuit exit in 250 mL samples for measuring the UV (220 nm) absorption. The analytical samples were named as follows: S1, S2, S3, and S4, in the order of their collection. Values are presented as the mean ± standard error for the six independent experiments. *P* < 0.05: a vs. e; a vs. i; a vs. m; b vs. f; b vs. j; b vs. m; c vs. g; c vs. k; c vs. o; d vs. h; d vs. l; d vs. p; o vs. g; o vs. h; p vs. g; p vs. l. Abbreviations: Gamma IR; PSu dialyzer [NV-15X (Toray Industries, Inc.)]; Autoclave; PSu dialyzer [RENAK-PS1.6 (Kawasumi Laboratories, Inc.)]; In-line steam; PSu dialyzer [FX-140J (Fresenius Medical Care)]; CTA; non-PSu CTA membrane dialyzer [FB-150U(NIPRO)].

**Fig. 2:**
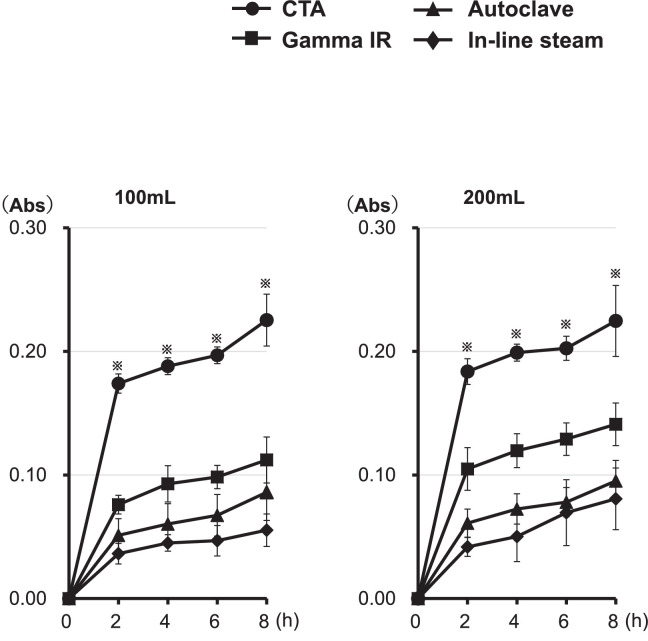
Effects of circulation duration and flow rate of saline on the *in vitro* elution of the unidentified substances from the primed dialyzers.

**Fig. 3:**
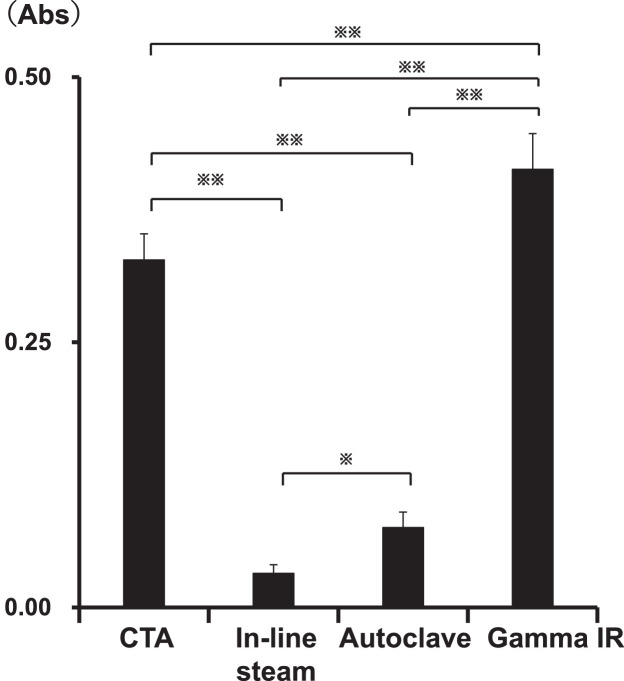
Profiles of the *in vitro* elution of unidentified substances from the primed dialyzers incubated with saline for 24 h without circulation.

**Fig. 4:**
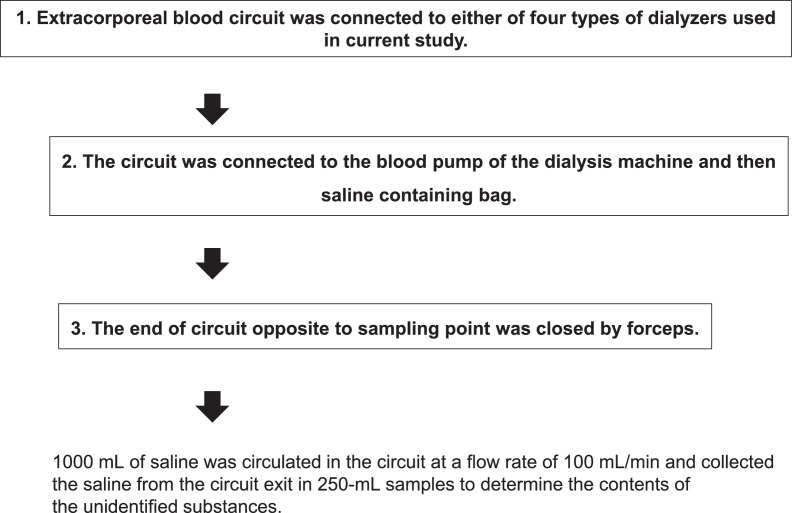

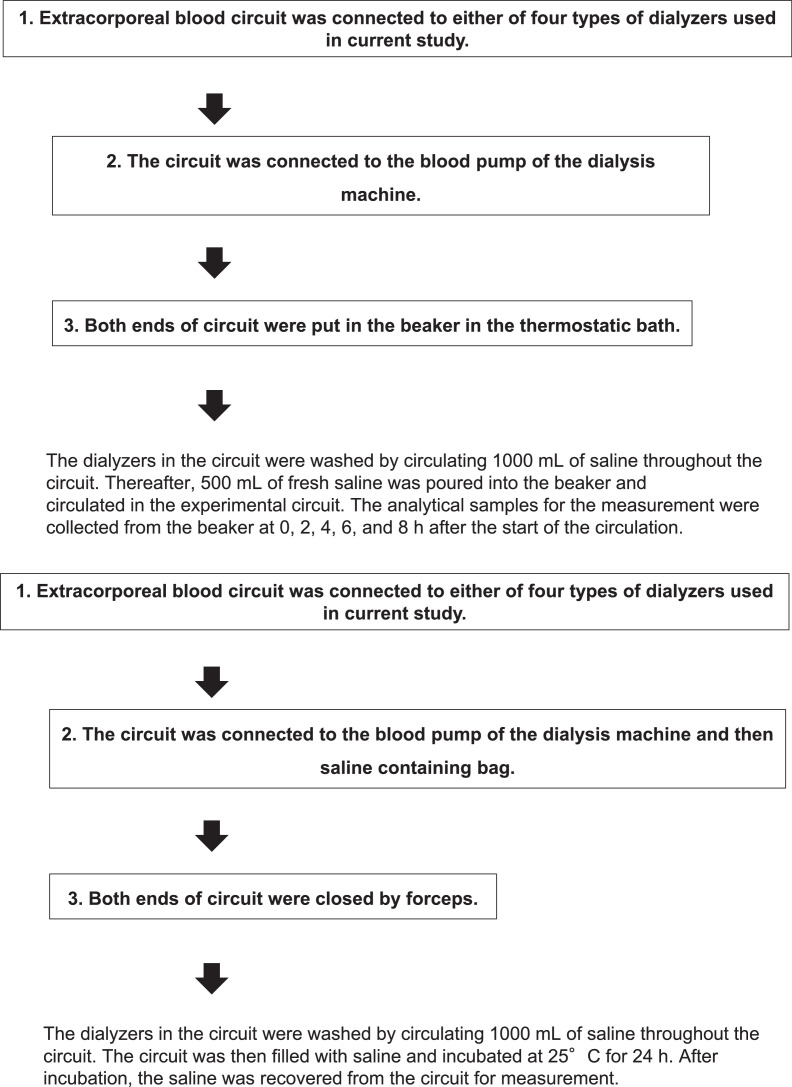
Schematic illustrations of procedures to build the experimental circuits used in experiments in Figs. 1 (a), 2 (b), and 3 (c).

Each primed dialyzer was set in the circuit described in Fig. 1B of the co-submitted article [1]. Saline was then circulated in the experimental circuit at a flow rate of 100 mL/min (left panel) or 200 mL/min (right panel). At the indicated time points after the start of circulation, the concentration of the unidentified substances in the beaker was determined as per the UV (220 nm) absorption. The values have been presented as the mean ± standard error for the six independent experiments. **^※^** *P* < 0.05. Abbreviations: Gamma IR; PSu dialyzer [NV-15X (Toray Industries, Inc.)]; Autoclave; PSu dialyzer [RENAK-PS1.6 (Kawasumi Laboratories, Inc.)]; In-line steam; PSu dialyzer [FX-140J (Fresenius Medical Care)]; CTA; non-PSu CTA membrane dialyzer [FB-150U(NIPRO)].

Each primed dialyzer was set in the circuit described in Fig. 1C of the co-submitted article [1] and was then incubated with saline at 25 °C for 24 h without circulation. After incubation, the saline was recovered for measuring the UV (220 nm) absorption. Values are presented as mean ± standard error for the six independent experiments. **^※^** *P* < 0.05, **^※※^** *P* < 0.01. Abbreviations: Gamma IR; PSu dialyzer [NV-15X (Toray Industries, Inc.)]; Autoclave; PSu dialyzer [RENAK-PS1.6 (Kawasumi Laboratories, Inc.)]; In-line steam; PSu dialyzer [FX-140J (Fresenius Medical Care)]; CTA; non-PSu CTA membrane dialyzer [FB-150U(NIPRO)].

**Table 1:** *In vitro* elution levels of substances in the priming, the circulation, and the immersion experiments corresponding to each of the dialyzers tested.

	Priming with saline (Analytical samples S1)	Saline circulation at a flow rate of 200 mL/min for 8 h	Immersion with saline for 24 h
Cellulose triacetate membrane dialyzer	0.51 ± 0.11 abs	0.22 ± 0.03 abs	0.33 ± 0.02 abs
Polyvinylpyrrolidone (PSu) membrane (In-line stem)	0.01 ± 0.11 abs	0.08 ± 0.02 abs	0.03 ± 0.01 abs
PSu membrane dialyzer (Autoclave)	0.03 ± 0.00 abs	0.10 ± 0.02 abs	0.08 ± 0.01 abs
PSu membrane dialyzer (Gamma IR)	0.07 ± 0.02 abs	0.14 ± 0.02 abs	0.41 ± 0.03 abs

**Table 2:** Raw data related to Fig. 1.

CTA; non-PSu CTA membrane dialyzer [FB-150U(NIPRO)]						
Sample name	abs (Experiment1)	abs (Experiment2)	abs (Experiment3)	abs (Experiment4)	abs (Experiment5)	abs (Experiment6)
S1	0.52	0.56	0.39	0.47	0.70	0.45
S2	0.21	0.17	0.17	0.21	0.19	0.18
S3	0.13	0.12	0.15	0.13	0.13	0.12
S4	0.10	0.10	0.09	0.11	0.10	0.09


In-line steam; PSu dialyzer [FX-140J (Fresenius Medical Care)]						
Sample name	abs (Experiment1)	abs (Experiment2)	abs (Experiment3)	abs (Experiment4)	abs (Experiment5)	abs (Experiment6)
S1	0.00	0.02	0.02	0.00	0.00	0.00
S2	0.00	0.02	0.02	0.00	0.00	0.00
S3	0.00	0.01	0.01	0.00	0.00	0.00
S4	0.00	0.01	0.00	0.01	0.00	0.00


Autoclave; PSu dialyzer [RENAK-PS1.6 (Kawasumi Laboratories, Inc.)]						
Sample name	abs (Experiment1)	abs (Experiment2)	abs (Experiment3)	abs (Experiment4)	abs (Experiment5)	abs (Experiment6)
S1	0.05	0.10	0.06	0.09	0.07	0.07
S2	0.02	0.02	0.03	0.07	0.03	0.05
S3	0.01	0.01	0.01	0.03	0.04	0.04
S4	0.01	0.01	0.01	0.03	0.03	0.04


Gamma IR; PSu dialyzer [NV-15X (Toray Industries, Inc.)]						
Sample name	abs (Experiment1)	abs (Experiment2)	abs (Experiment3)	abs (Experiment4)	abs (Experiment5)	abs (Experiment6)
S1	0.02	0.02	0.03	0.02	0.02	0.03
S2	0.02	0.00	0.01	0.01	0.01	0.01
S3	0.01	0.00	0.01	0.01	0.01	0.01
S4	0.01	0.00	0.00	0.00	0.00	0.01

**Table 3:** Raw data related to Fig. 2 (Experiment with flow rate of 100 ml/min).

CTA; non-PSu CTA membrane dialyzer [FB-150U(NIPRO)]						
Circulation duration (h)	abs (Experiment1)	abs (Experiment2)	abs (Experiment3)	abs (Experiment4)	abs (Experiment5)	abs (Experiment6)
0	0.00	0.00	0.00	0.00	0.00	0.00
2	0.16	0.18	0.18	0.17	0.17	0.18
4	0.18	0.20	0.19	0.18	0.19	0.19
6	0.19	0.21	0.20	0.19	0.20	0.19
8	0.20	0.20	0.22	0.25	0.25	0.24


In-line steam; PSu dialyzer [FX-140J (Fresenius Medical Care)]						
Circulation duration (h)	abs (Experiment1)	abs (Experiment2)	abs (Experiment3)	abs (Experiment4)	abs (Experiment5)	abs (Experiment6)
0	0.00	0.00	0.00	0.00	0.00	0.00
2	0.04	0.03	0.05	0.03	0.03	0.04
4	0.05	0.04	0.05	0.05	0.05	0.03
6	0.04	0.04	0.03	0.05	0.07	0.05
8	0.05	0.05	0.04	0.06	0.08	0.05


Gamma IR; PSu dialyzer [NV-15X (Toray Industries, Inc.)]						
Circulation duration (h)	abs (Experiment1)	abs (Experiment2)	abs (Experiment3)	abs (Experiment4)	abs (Experiment5)	abs (Experiment6)
0	0.00	0.00	0.00	0.00	0.00	0.00
2	0.06	0.08	0.08	0.08	0.08	0.08
4	0.08	0.09	0.08	0.12	0.10	0.10
6	0.09	0.10	0.09	0.11	0.10	0.10
8	0.15	0.11	0.09	0.11	0.10	0.11


Autoclave; PSu dialyzer [RENAK-PS1.6 (Kawasumi Laboratories, Inc.)]						
Circulation duration (h)	abs (Experiment1)	abs (Experiment2)	abs (Experiment3)	abs (Experiment4)	abs (Experiment5)	abs (Experiment6)
0	0.00	0.00	0.00	0.00	0.00	0.00
2	0.03	0.05	0.06	0.07	0.05	0.06
4	0.04	0.05	0.07	0.09	0.05	0.06
6	0.04	0.06	0.09	0.08	0.06	0.07
8	0.05	0.07	0.10	0.11	0.11	0.08

**Table 4:** Raw data related to Fig. 2 (Experiment with flow rate of 200 ml/min).

CTA; non-PSu CTA membrane dialyzer [FB-150U(NIPRO)]						
Circulation duration (h)	abs (Experiment1)	abs (Experiment2)	abs (Experiment3)	abs (Experiment4)	abs (Experiment5)	abs (Experiment6)
0	0.00	0.00	0.00	0.00	0.00	0.00
2	0.17	0.19	0.19	0.20	0.18	0.18
4	0.19	0.21	0.20	0.21	0.20	0.19
6	0.19	0.21	0.21	0.21	0.20	0.20
8	0.22	0.26	0.26	0.21	0.20	0.21


In-line steam; PSu dialyzer [FX-140J (Fresenius Medical Care)]						
Circulation duration (h)	abs (Experiment1)	abs (Experiment2)	abs (Experiment3)	abs (Experiment4)	abs (Experiment5)	abs (Experiment6)
0	0.00	0.00	0.00	0.00	0.00	0.00
2	0.03	0.05	0.04	0.05	0.04	0.04
4	0.04	0.05	0.04	0.09	0.03	0.05
6	0.08	0.06	0.10	0.10	0.03	0.06
8	0.11	0.06	0.09	0.11	0.05	0.07


Gamma IR; PSu dialyzer [NV-15X (Toray Industries, Inc.)]						
Circulation duration (h)	abs (Experiment1)	abs (Experiment2)	abs (Experiment3)	abs (Experiment4)	abs (Experiment5)	abs (Experiment6)
0	0.00	0.00	0.00	0.00	0.00	0.00
2	0.14	0.10	0.10	0.09	0.11	0.10
4	0.13	0.10	0.13	0.11	0.11	0.13
6	0.15	0.11	0.14	0.12	0.12	0.14
8	0.15	0.13	0.14	0.17	0.12	0.14


Autoclave; PSu dialyzer [RENAK-PS1.6 (Kawasumi Laboratories, Inc.)]						
Circulation duration (h)	abs (Experiment1)	abs (Experiment2)	abs (Experiment3)	abs (Experiment4)	abs (Experiment5)	abs (Experiment6)
0	0.00	0.00	0.00	0.00	0.00	0.00
2	0.07	0.07	0.07	0.06	0.05	0.05
4	0.09	0.08	0.08	0.06	0.07	0.06
6	0.08	0.09	0.08	0.06	0.09	0.07
8	0.08	0.12	0.11	0.08	0.08	0.10

**Table 5:** Raw data related to Fig. 4.

Dialyzer	abs (Experiment1)	abs (Experiment2)	abs (Experiment3)	abs (Experiment4)	abs (Experiment5)	abs (Experiment6)
Gamma IR	0.41	0.37	0.47	0.42	0.40	0.41
CTA	0.31	0.32	0.32	0.34	0.31	0.37
Autoclave	0.06	0.07	0.07	0.08	0.07	0.10
In-line steam	0.03	0.03	0.03	0.03	0.04	0.04

## 2 Experimental Design, Substances, and Methods

### 2.1 Dialyzers

This study included three types of PSu membrane dialyzers and one non-PSu membrane dialyzer. The PSu membrane dialyzers were sterilized with either gamma irradiation [NV-15X (Toray Industries, Inc.)], autoclaving [RENAK-PS1.6 (Kawasumi Laboratories, Inc.)], or in-line steam [FX-140J (Fresenius Medical Care)] methods. The non-PSu membrane dialyzer was a cellulose triacetate (CTA) membrane dialyzer [FB-150U (NIPRO)] and was sterilized with either gamma irradiation. Surface areas of PSu membrane dialyzer [NV-15X (Toray Industries, Inc.)], PSu membrane dialyzer [RENAK-PS1.6 (Kawasumi Laboratories, Inc.)], PSu membrane dialyzer [FX-140J (Fresenius Medical Care)], and non-PSu membrane dialyzer [FB-150U (NIPRO)] are 1.5 m^2^, 1.6 m^2^, 1.4 m^2^, and 1.5 m^2^, respectively.

### 2.2 Preparation of the experimental circuits

To test the effects of the priming process on the *in vitro* elution of unidentified substances from the PSu and non-PSu dialyzer membranes, the experimental circuit illustrated in Fig. 1A of the co-published article [1] was built as following (Fig. 4a). Extracorporeal blood circuit containing air traps (Kawasumi Laboratories, Inc.) was first connected to either of above-mentioned dialyzers. Next, the circuit was connected to the blood pump of the dialysis machine (Toray Medical Co., Ltd.) and then saline (0.9% NaCl) containing bag (Toray Medical Co., Ltd.). Finally, the end of circuit opposite to sampling point was closed by forceps. Thereafter, we circulated 1000 mL of saline in the circuit at a flow rate of 100 mL/min and collected the saline from the circuit exit in 250 mL samples to determine the contents of the unidentified substances.

To examine the effects of the circulation conditions on the *in vitro* elution of the unidentified substances from the dialyzer membranes, the experimental circuit illustrated in Fig. 1B of the co-published article [1] was built as following (Fig. 4b). Extracorporeal blood circuit containing air traps (Kawasumi Laboratories, Inc.) was first connected to either of above-mentioned dialyzers. Next, the circuit was connected to the blood pump of the dialysis machine (Toray Medical Co., Ltd.). Finally, both ends of circuit were put in the beaker. The dialyzers in the circuit were washed by circulating 1000 mL of saline throughout the circuit. Thereafter, 500 mL of fresh saline (37 °C) was poured into the beaker and circulated in the experimental circuit at a flow rate of 100 mL/min or 200 mL/min. The beaker was put in the thermostatic bath to keep the temperature of saline at 37 °C during the circulation. The analytical samples for the measurement were collected from the beaker at 0, 2, 4, 6, and 8 h after the start of the circulation.

To examine the effect of incubation of the dialyzer membranes with saline on the elution of unidentified substances from the primed dialyzers, the experimental circuit illustrated in Fig. 1C of the co-published article [1] was built as following (Fig. 4c). Extracorporeal blood circuit containing air traps (Kawasumi Laboratories, Inc.) was first connected to either of above-mentioned dialyzers. Next, the circuit was connected to the blood pump of the dialysis machine (Toray Medical Co., Ltd.) and then saline (0.9% NaCl) containing bag (Toray Medical Co., Ltd.). Finally, both ends of circuit were closed by forceps. The dialyzers in the circuit were washed by circulating 1000 mL of saline throughout the circuit. The circuit was then filled with saline and incubated at 25 °C for 24 h. After incubation, the saline was recovered from the circuit for measurement.

### 2.3 Measurement of the UV absorption of the eluates

The UV (220 nm) absorption of the eluates was determined using a spectrophotometer (Hitachi; U-5100). One ml of each analytical sample was put in quartz cell for measurement of the UV absorption.

### 2.4 Statistical analyze

The statistical significance was calculated using Statcel4 software (The Publisher OMS Ltd. Japan). First, the normality of the distribution was evaluated by comparing the results from the experiments using the four different dialyzers. If normal distribution was confirmed, the Tukey–Kramer method was used for multigroup analysis (Fig. 3). If normal distribution was not confirmed, the multigroup analysis was performed using the Steel–Dwass method (Figs. 1 and 2).

## Ethics Statement

NA.

## CRediT authorship contribution statement

**Yoshinori Sato:** Visualization, Formal analysis, Investigation. **Hayato Horiuchi:** Investigation. **Shinji Fukasawa:** Investigation. **Shingo Takesawa:** Formal analysis. **Jun Hirayama:** Formal analysis, Writing – original draft.

## Declaration of Competing Interest

The authors declare that they have no known competing financial interests or personal relationships that have or could be perceived to have influenced the work reported in this article.
